# Transfer of cardiomyocyte-derived extracellular vesicles to neighboring cardiac cells requires tunneling nanotubes during heart development

**DOI:** 10.7150/thno.91604

**Published:** 2024-06-17

**Authors:** Ting Chen, Ditte Gry Ellman, Shu Fang, Sara Thornby Bak, Mikkel Ørnfeldt Nørgård, Per Svenningsen, Ditte Caroline Andersen

**Affiliations:** 1 Andersen Group, Department of Clinical Biochemistry, Odense University Hospital, Odense, Denmark.; 2 Institute of Clinical Research, University of Southern Denmark, Odense, Denmark.; 3 Department of Urology, Children's Hospital, Zhejiang University School of Medicine, National Clinical Research Center for Child Health, Hangzhou, China.; 4 Department of Molecular Medicine, Unit of Cardiovascular and Renal Research, University of Southern Denmark, Odense, Denmark.

**Keywords:** cardiomyocyte, extracellular vesicles, exosomes, tunneling nanotubes, heart

## Abstract

**Rationale:** Extracellular vesicles (EVs) are thought to mediate intercellular communication during development and disease. Yet, biological insight to intercellular EV transfer remains elusive, also in the heart, and is technically challenging to demonstrate. Here, we aimed to investigate biological transfer of cardiomyocyte-derived EVs in the neonatal heart.

**Methods:** We exploited CD9 as a marker of EVs, and generated two lines of cardiomyocyte specific EV reporter mice: *Tnnt2-Cre*; double-floxed inverted *CD9/EGFP* and *αMHC-MerCreMer;* double-floxed inverted* CD9/EGFP*. The two mouse lines were utilized to determine whether developing cardiomyocytes transfer EVs to other cardiac cells (non-myocytes and cardiomyocytes) *in vitro* and *in vivo* and investigate the intercellular transport pathway of cardiomyocyte-derived EVs.

**Results:** Genetic tagging of cardiomyocytes was confirmed in both reporter mouse lines and proof of concept in the postnatal heart showed that, a fraction of EGFP^+^/MYH1^-^ non-myocytes exist firmly demonstrating *in vivo* cardiomyocyte-derived EV transfer. However, two sets of direct and indirect EGFP*^+/-^* cardiac cell co-cultures showed that cardiomyocyte-derived EGFP^+^ EV transfer requires cell-cell contact and that uptake of EGFP^+^ EVs from the medium is limited. The same was observed when co-cultiring with mouse macrophages. Further mechanistic insight showed that cardiomyocyte EV transfer occurs through type I tunneling nanotubes.

**Conclusion:** While the current notion assumes that EVs are transferred through secretion to the surroundings, our data show that cardiomyocyte-derived EV transfer in the developing heart occurs through nanotubes between neighboring cells. Whether these data are fundamental and relate to adult hearts and other organs remains to be determined, but they imply that the normal developmental process of EV transfer goes through cell-cell contact rather than through the extracellular compartment.

## Introduction

Extracellular vesicles (EVs) are lipid-bound vesicles that carry complex biological information 1, 2. They transfer bioactive substances including proteins, lipids, and nucleic acids to target cells, and play key roles in intercellular communication 3, 4. EVs include, but are not limited to, microvesicles and exosomes 5, but should be referred to as EVs according to the international guideline MISEV2018 6, when the subcellular origin of the vesicles is lacking. The current model for intercellular EV signaling proposes that EVs are secreted either via the outward budding of the plasma membrane (microvesicles) 5 or via fusion of multivesicular bodies (MVBs) to the plasma membrane and then excreted by exocytosis (exosomes) 7, 8 into the surroundings, whereafter they may be taken up by other cells either locally or at a distance 9, 10. EVs are suggested to interact with recipient cells through multiple mechanisms including ligand-receptor binding, fusion with the plasma membrane, and endocytic pathways such as phagocytosis and endocytosis 11-13. Cardiomyocyte-derived EVs have been reported to play an important role in cardiac development 14, cardiac homeostasis 15, 16, and in the differentiation of multipotent stem cells towards cardiac cell types 17, through intercellular communication. However, in line with the EV field in general, the underlying mechanisms of cardiomyocyte EVs secretion and uptake by other cells are poorly understood. In fact, many studies assume that cardiomyocyte-derived EVs are delivered to neighboring cardiac cells in a paracrine manner by default. However, the biological function of cardiomyocyte-derived EVs is often ascribed by using a much higher concentration of cardiomyocyte-derived EVs for *in vitro* experiments than *in vivo* 18-20. Due to the higher concentrations of cardiomyocyte-derived EVs, events that are considered unlikely to occur *in vivo* become feasible *in vitro* 21. Collectively, these observations question to what degree cardiomyocyte-derived EVs exist *in vivo* and, furthermore, to what degree they communicate biological entities at levels capable of altering the behavior of recipient cells.

For tracking EVs in mouse models, genetic labeling of EVs has been considered the most reliable method, and typically involves the fusion of the EV-enriched tetraspanins, such as CD9 and CD63, with reporter proteins like enhanced green fluorescent protein (EGFP) 22-25. Yet, most tracing studies hereto have merely demonstrated the EV uptake *in vitro* 23-25. Recently, different transgenic endogenous EV reporter mice have been developed and which exploit genetic labeling of EV bound tetraspanins, but while these models enable *in vivo* biodistribution of cardiac EVs to be traced, they provide poor insight into subcellular localization and how intercellular EV transfer occurs 22, 23.

Here, we developed a new genetic approach using an extravesicular EGFP-tag and generated two mouse models to track cardiomyocyte-derived EVs *in vitro* and *in vivo*, and by proof-of-concept investigated how cardiomyocyte-derived EVs transfer between cardiac cells in the postnatal heart. We hypothesized that this may aid in new biological insight into the mechanism of EV transfer and the future use of EVs in various fields of cardiac research and medicine.

## Results

### Cardiomyocyte-derived EV EGFP-reporter mice as novel tools to study cardiomyocyte-derived EVs in the developing postnatal heart

**Figure 1:**
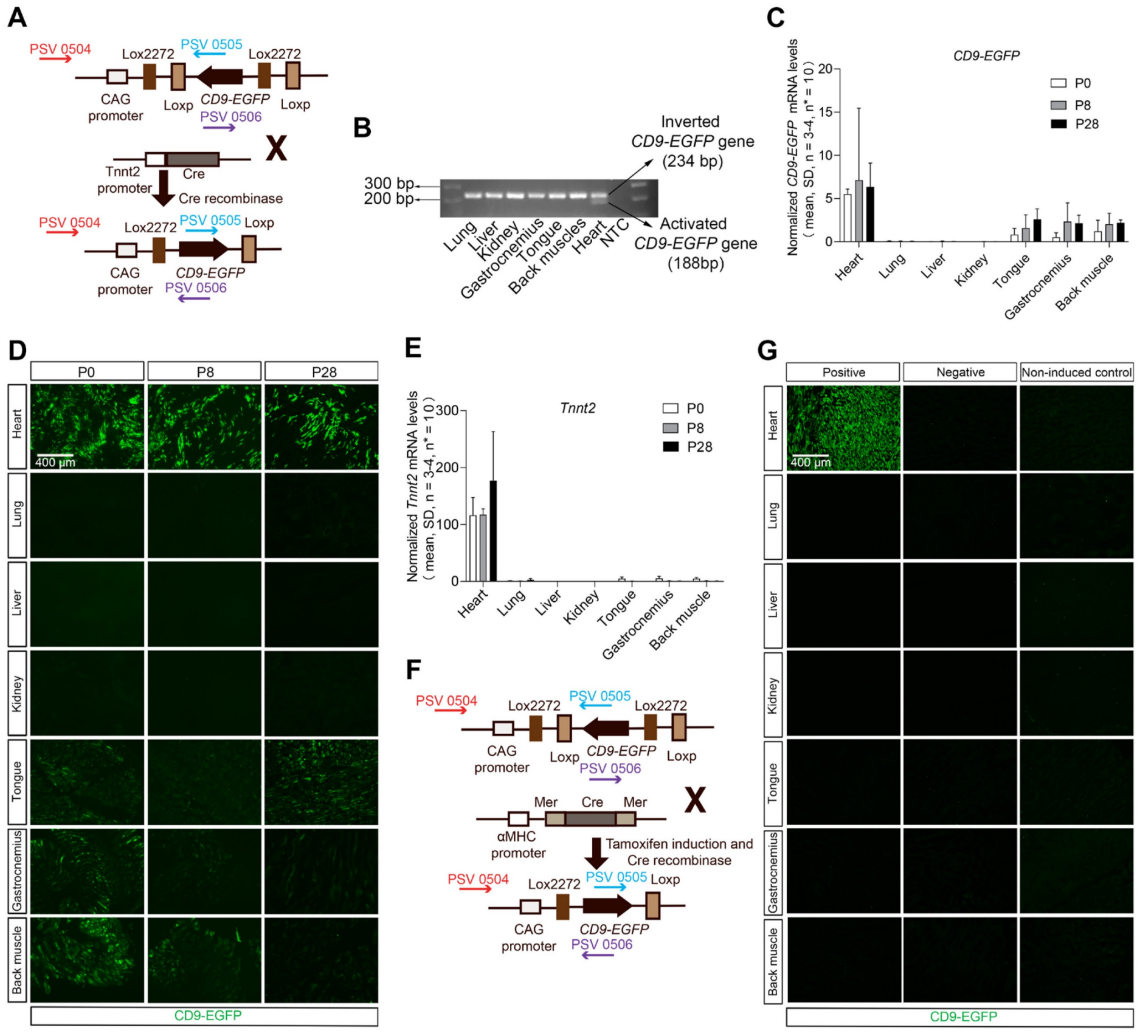
** Establishment and validation of inducible cardiomyocyte-derived EV reporter mouse lines.** (**A**) Breeding strategy used to cross double-floxed inverted *CD9/EGFP* mice with *Tnnt2-Cre* mice to specifically activate the *CD9-EGFP* gene in cardiomyocytes. Arrows indicate primers designed to differentiate between inverted and activated* CD9-EGFP* genes (PSV 0504, 0505, and 0506). (**B**) Agarose gel electrophoresis for PCR products of inverted *CD9-EGFP* gene (234 bp) and activated *CD9-EGFP* gene (188 bp) (n = 3). (**C**) Relative mRNA expression of* CD9-EGFP* in various organs from P0, P8, and P28 *Tnnt2-Cre*; double-floxed inverted *CD9/EGFP ^+^* mice. Data were normalized against multiple stably expressed endogenous controls (*β-Acti*n and *RPL13A*) as determined by the qbase^+^ platform. (**D**) Direct fluorescence of different organ sections from P0, P8, and P28 *Tnnt2-Cre*; double-floxed inverted *CD9/EGFP* ^+^ mice. EGFP signals (green) were observed in the heart and skeletal muscles (tongue, gastrocnemius, and back muscle) (n = 3). (**E**) Relative mRNA expression of *Tnnt2* in various organs from P0, P8, and P28* Tnnt2-Cre*; double-floxed inverted *CD9/EGFP* ^+^ mice. Data were normalized against multiple stably expressed endogenous controls (*β-Actin* and *RPL13A*) as determined by the qbase^+^ platform. (**F**) Breeding strategy used to cross double-floxed inverted *CD9/EGFP* mice with *αMHC-MerCreMer* mice to conditionally activate the *CD9-EGFP* gene in cardiomyocytes. Arrows indicate primers designed to differentiate between inverted and activated* CD9-EGFP* genes (PSV 0504, 0505, and 0506). (**G**) Immunofluorescence staining of different organ sections from P28 *αMHC-MerCreMer*; double-floxed inverted *CD9-EGFP* mice. EGFP signals (green) were only present in the heart in positive mice (n = 3). Positive: P28 *αMHC-MerCreMer*; double-floxed inverted *CD9-EGFP ^+^* mice, Negative: P28 *αMHC-MerCreMer*; double-floxed inverted *CD9-EGFP ^-^* mice, Non-induced control: non-induced P28 *αMHC-MerCreMer*; double-floxed inverted *CD9-EGFP* mice.

Recently, we developed a double-floxed inverted *CD9/EGFP* mouse in which CD9-EGFP expression is activated upon crossbreeding with Cre recombinase mice 26. In that genetic system, we have shown by tunable resistive pulse sensing that CD9-EGFP expression does not alter the size or abundance of EVs, and that EGFP-labeled EVs co-express EV markers such as TSG101 and ALIX 26. Taking advantage of this genetic system we here developed an approach to visualize cardiomyocyte-derived EVs, and thus generated heterozygous* Tnnt2-Cre*; double-floxed inverted *CD9/EGFP* mouse through crossing the *Tnnt2-Cre* mice with double-floxed inverted *CD9/EGFP* mice (Figure 1A). The cardiomyocyte-specific recombination of the LoxP sites was identified by PCR and present in 22.0% of pups (60/273, 49 litters). Throughout the remainder of the study, we used single heterozygous and wild-type littermates as controls. Cre-mediated recombination was detected by PCR in the heart, but not in other organs (Figure 1B). The expression of the transgene was verified by qRT-PCR, where activated CD9-EGFP transcripts were detected in the heart but also to some extent in skeletal muscles (Figure 1C), which was consistent with the CD9-EGFP fusion protein expression pattern observed (Figure 1D). As expected from the heterozygous mouse design, not all cardiomyocytes were CD9-EGFP^+^ (Figure 1D), but colocalization of EGFP and CD9 *in vivo* and *in vivo* verified that CD9-EGFP was a reliable marker of CD9 (Figure S1). The CD9-EGFP expression observed in skeletal muscle correlated to residual *Tnnt2* activity (Figure 1E). Moreover, no CD9-EGFP fusion protein was observed in the lung, liver, and kidney (Figure 1D) likely indicating that cardiomyocyte-derived EVs do not randomly distribute to other organs. Importantly, we did not observe any leakage of EGFP in littermate controls (Figure S1). To test whether these results were reproducible in another mouse model with high Cre activation of cardiomyocyte CD9-EGFP, we crossed the well-known and cardiomyocyte-specific Cre mouse line *αMHC-MerCreMer* 27-29 with our double-floxed inverted *CD9/EGFP* mice to generate *αMHC-MerCreMer;* double-floxed inverted* CD9/EGFP* mice (Figure 1F). Like previous studies 30-32, the administration of tamoxifen to pregnant mice induced dystocia during the parturition time (Table S1). We overcame this challenge by injecting tamoxifen after birth and obtained P28 *αMHC-MerCreMer;* double-floxed inverted* CD9/EGFP* mice expressing EGFP specifically in the heart (Figure 1G). A larger fraction of cells in the P28 hearts expressed EGFP in *αMHC-MerCreMer;* double-floxed inverted* CD9/EGFP* mice, as compared to *Tnnt2-Cre*; double-floxed inverted *CD9/EGFP* mouse hearts, and the expression was restricted to hearts (Figure 1G). Thus, whereas the* Tnnt2-Cre*; double-floxed inverted *CD9/EGFP* represent a novel genetic tool to study cardiomyocyte-derived EVs during heart development, the *αMHC-MerCreMer;* double-floxed inverted* CD9/EGFP* mouse may be a better alternative when studying EVs in the adult heart.

### Cardiomyocyte-derived EVs are taken up *in vivo* locally in the heart only

**Figure 2:**
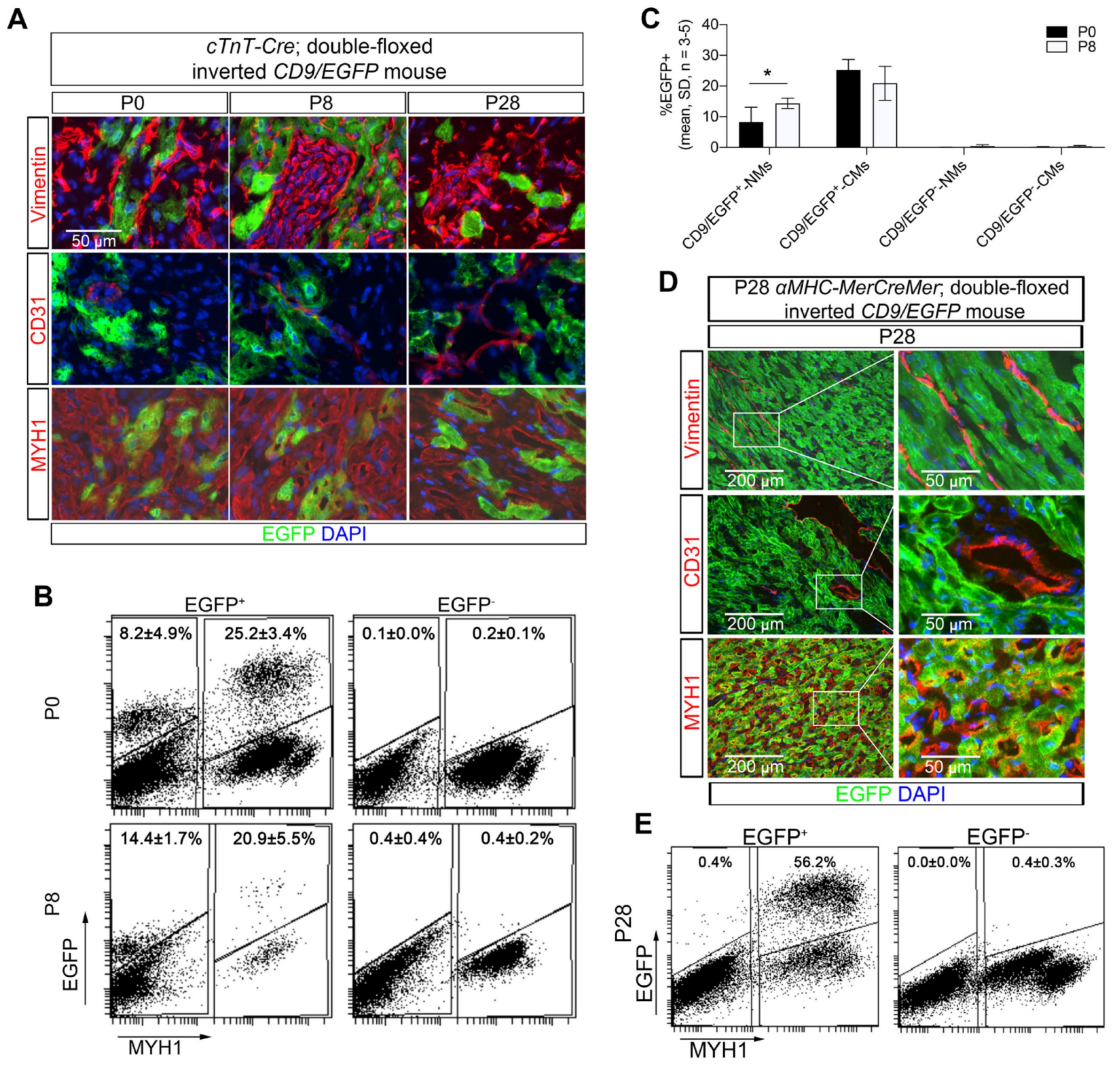
** Neonatal cardiomyocyte-derived EVs are transferred to non-myocytes *in vivo.*** (**A**) Immunofluorescence staining of heart sections from P0, P8, and P28 *Tnnt2-Cre*; double-floxed inverted *CD9/EGFP ^+^* mice. EGFP signals (green) were observed in cardiomyocytes (MYH1^+^), but not in fibroblasts (Vimentin^+^) and endothelial cells (CD31^+^). Nuclei are counterstained with DAPI (blue), and representative images are shown (n = 3). (**B-C**) Representative flow cytometry plots of EGFP and MYH1 expression in cardiac cells isolated from hearts at P0 and P8 for *Tnnt2-Cre*; double-floxed inverted *CD9/EGFP ^+^ and ^-^* mice (n = 3-5). Gates were defined from negative littermates and used for determining the indicated mean percentages of subpopulations. NMs: Non-myocytes, CMs: Cardiomyocytes. (**D**) Immunofluorescence staining of heart sections from P28 *αMHC-MerCreMer;* double-floxed inverted* CD9/EGFP ^+^* mice. EGFP signals (green) were observed in cardiomyocytes (MYH1^+^), but not in fibroblasts (Vimentin^+^) and endothelial cells (CD31^+^). Nuclei are counterstained with DAPI (blue), and representative images are shown (n = 3). (**E**) Representative flow cytometry plots of EGFP and MYH1 expression in cardiac cells isolated from hearts at P28 *αMHC-MerCreMer;* double-floxed inverted* CD9/EGFP ^+^ and^ -^* mice (n = 1-3). Gates were defined from negative littermates and used for determining the indicated mean percentages of subpopulations. Data are shown as the mean ± SD. Two-way ANOVA with Sidak multiple comparison test. * p < 0.05.

To show proof-of-concept of our new cardiomyocyte-derived EV genetic approach, we evaluated subcellular distribution of cardiomyocyte-derived EVs in the heart. Indeed, transfer of cardiomyocyte-derived EVs to cardiac fibroblasts and endothelial cells has been reported to occur as part of intercellular communication pathways 33, 34. Co-localization of EGFP and markers for cardiomyocytes (MYH1), fibroblasts (Vimentin), and endothelial cells (CD31) were herein investigated in postnatal (P0, P8, and P28) *Tnnt2-Cre*; double-floxed inverted *CD9/EGFP* mouse hearts by immunofluorescence staining (Figure 2A). Overall, we observed that only a fraction of the cardiomyocytes in the *Tnnt2-Cre*; double-floxed inverted *CD9/EGFP* hearts expressed EGFP regardless of developmental stage (Figure 2A). Because cardiomyocyte-derived EVs have been shown to be transferred to non-cardiomyocyte cardiac cells *in vivo*, it was expected that non-cardiomyocyte cardiac cells that were EGFP-positive would also be detected. However, unexpectedly, EGFP signals were only observed in cardiomyocytes, whereas fibroblasts and endothelial cells appeared negative (Figure 2A) despite antibody amplification of the EGFP signal (Figure S2). To avoid poor EGFP detection limits using immunocytochemistry, we further enhanced EGFP detection by high-resolution flow cytometry 35-37. As such, we isolated from postnatal hearts the heterogenous pool of cells including cardiomyocytes (MYH1^+^), fibroblasts, and endothelial cells (MYH1^-^) from *Tnnt2-Cre*; double-floxed inverted *CD9/EGFP* mouse hearts and subjected them to flow cytometric analysis (Figure 2B-C). By this, we identified a fraction of EGFP^+^/MYH1^-^ non-myocytes that was absent in EGFP negative littermates (Figure 2B-C). Interestingly, there was no significant difference in the percentage of EGFP^+^ cardiomyocytes in the heart between the P0 and P8* Tnnt2-Cre*; double-floxed inverted *CD9/EGFP^+^* mice. In contrast, a modest, but significant increase in the percentage of EGFP^+^ non-myocytes were observed at P8 as compared to P0 (Figure 2B-C). Also, in the *αMHC-MerCreMer;* double-floxed inverted* CD9/EGFP* mice at postnatal day P28 (Figure 2D-E) we found that a small percentage of non-myocytes was EGFP^+^/MYH1^-^. These data thus firmly track that non-myocytes in the heart take up EVs derived from cardiomyocytes *in vivo* at different postnatal developmental stages and confirm the useability of our *αMHC-MerCreMer- or Tnnt2-Cre*; double-floxed inverted *CD9/EGFP* models especially *in vivo*.

### Neonatal cardiomyocyte-derived EVs are transferred by direct cell-to-cell contact

**Figure 3:**
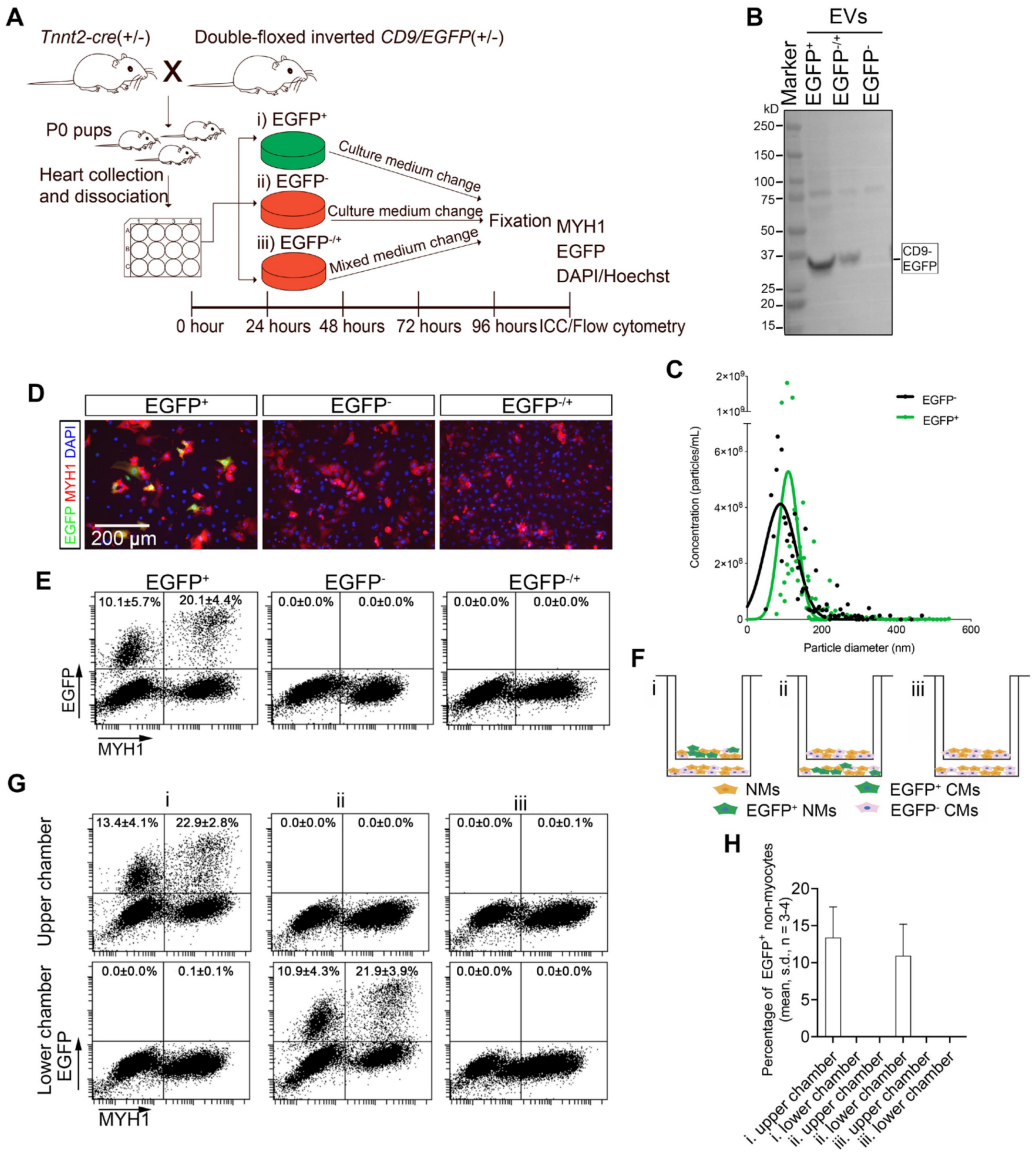
** Neonatal cardiomyocyte-derived EV transfer requires direct cell-to-cell contact.** (**A-E**) (**A**) Schematic study design with three groups of primary neonatal cardiac cells i) EGFP^+^ (*Tnnt2-Cre*; double-floxed inverted *CD9/EGFP^+^*), ii) EGFP^-^ (*Tnnt2-Cre*; double-floxed inverted *CD9/EGFP^-^), and iii)* EGFP^-/+^ (*Tnnt2-Cre*; double-floxed inverted *CD9/EGFP^-^* with EGFP^+^ conditioned medium)*.* (**B**) Western blotting of precipitated EVs from cell culture media. (**C**) Particle concentration and size distribution in conditioned medium from *Tnnt2-Cre*; double-floxed inverted *CD9/EGFP* positive (EGFP^+^) and their EGFP^-^ littermates (EGFP^-^). At 96 h, cells were fixed and further analyzed by (**D**) immunofluorescence (EGFP (green), MYH1^+^ (red), and DAPI (blue)) and (**E**) and flow cytometry for MYH1 and EGFP. (**F-H**) (**F**) Schematic diagram of transwell co-culture system illustrating upper- and lower chambers with the three experimental groups i) EGFP^+^/ EGFP^-^, ii) EGFP^-^/ EGFP^+^, and iii) EGFP^-^/ EGFP^-^ primary neonatal cardiac cells. After 72 h, (**G**) cultures in both chambers were analyzed by flow cytometry for EGFP and MYH1, and (**H**) the percentages of EGFP^+^ non-myocytes were determined. For statistical analysis, we used one-way ANOVA followed by Dunnett's post-test (n = 3-4). NMs: Non-myocytes, CMs: Cardiomyocytes.

It is generally presumed that EVs are transferred to neighboring cells via paracrine mechanisms, but evidence has been limited to the use of isolated EVs at non-physiological concentrations. Therefore, we speculated whether our *Tnnt2-Cre*; double-floxed inverted *CD9/EGFP* model could be used to finally confirm that cardiomyocyte-derived EVs can be efficiently taken up by neighboring cardiomyocytes and non-myocytes from the surroundings without a prior EV isolation step and use of biological EV concentrations. We therefore designed an assay where conditioned medium from *Tnnt2-Cre*; double-floxed inverted *CD9/EGFP* positive (EGFP^+^) cell cultures were added to *Tnnt2-Cre*; double-floxed inverted *CD9/EGFP* negative (EGFP^-^) cardiac cell cultures (referred to as EGFP^-/+^), refreshing the conditioned medium every day to ensure a vast number of EGFP^+^ EVs available (Figure 3A). Initially, we confirmed by precipitation and WB that the conditioned medium from EGFP^+^ and EGFP*^-/+^* cultures indeed contained EGFP^+^ EVs whereas EGFP^-^ cultures lacked EGFP^+^ EVs (Figure 3B). Notably, *Tnnt2-Cre*; double-floxed inverted *CD9/EGFP* positive (EGFP^+^) and their negative (EGFP^-^) littermates showed similar EV concentration and size distribution (Figure 3C), in the expected range of EVs 38 suggesting that neonatal cardiomyocyte-derived EVs do not exhibit any remarkable changes upon EGFP labeling. Thus, as expected *Tnnt2-Cre*; double-floxed inverted *CD9/EGFP* positive neonatal cardiomyocytes secrete EGFP-labeled EVs into the culture medium. We next tracked EGFP^+^ EV uptake in neonatal EGFP^-^ cultures supplemented with EGFP^+^ conditioned medium using immunofluorescence and flow cytometry (Figures 3D-E, and S3). In agreement with the *in vivo* data (Figure 2), the neonatal EGFP^+^ cardiac cultures represented EGFP^+^ cardiomyocytes (20.1 ± 4.4%; mean, SD, n = 4) and EGFP^+^ non-myocytes (10.1 ± 5.7%; mean, SD, n = 4) (Figures 3D-E). The EGFP signal was higher in cardiomyocytes than in non-myocytes in line with the cardiomyocytes being the origin of EGFP^+^ EV production (Figure 3E), but an intermediate population of EGFP^+^ cardiomyocytes appeared as well (Figure 3E) likely reflecting that non transgenic neonatal cardiomyocytes also take up EGFP^+^ EVs from neighboring cardiomyocytes. However, we did not observe any EGFP signal in EGFP^-^ cultures enriched with EGFP^+^ conditioned medium (Figures 3D-E). This surprisingly indicates that the transfer of neonatal cardiomyocyte-derived EGFP-labeled EVs to neighboring neonatal cardiac cells possibly is mediated by direct cell-to-cell contact and not by uptake directly from the surroundings (media) as proposed hereto 18-20.

To further verify these results, we designed another trans-well co-culture assay between EGFP^+^ and EGFP^-^ neonatal cardiac cells (Figure 3F), where the cells are not in contact, but share their medium directly. Whereas 13.4 ± 4.1% and 10.9 ± 4.3% (mean, SD, n = 3-4) EGFP^+^ non-myocytes were present in EGFP^+^ cardiac cultures in upper and lower chambers, respectively, no EGFP signal was detected in co-cultured EGFP^-^ cardiac cells regardless the position in chambers (Figures 3F-H). Thus, both neonatal cardiac co-culture designs suggest that cardiomyocyte-derived EVs are transferred by a direct cell-to-cell contact mechanism and not through the extracellular compartment.

### Cardiomyocyte-derived EVs are transferred from neonatal cardiomyocytes to neighboring cells via tunneling nanotubes

We then speculated on how the neonatal cardiomyocytes via cell-cell contact mediate EV transfer to neighboring cardiac cells. The direct cell-to-cell contact mechanisms mainly include gap junctions and tunneling nanotubes (TNTs) 39. Previous studies have shown that isolated tumor EVs can be transferred via TNTs 40, 41. We therefore investigated whether TNTs were involved in the transfer of cardiomyocyte-derived EVs.

**Figure 4:**
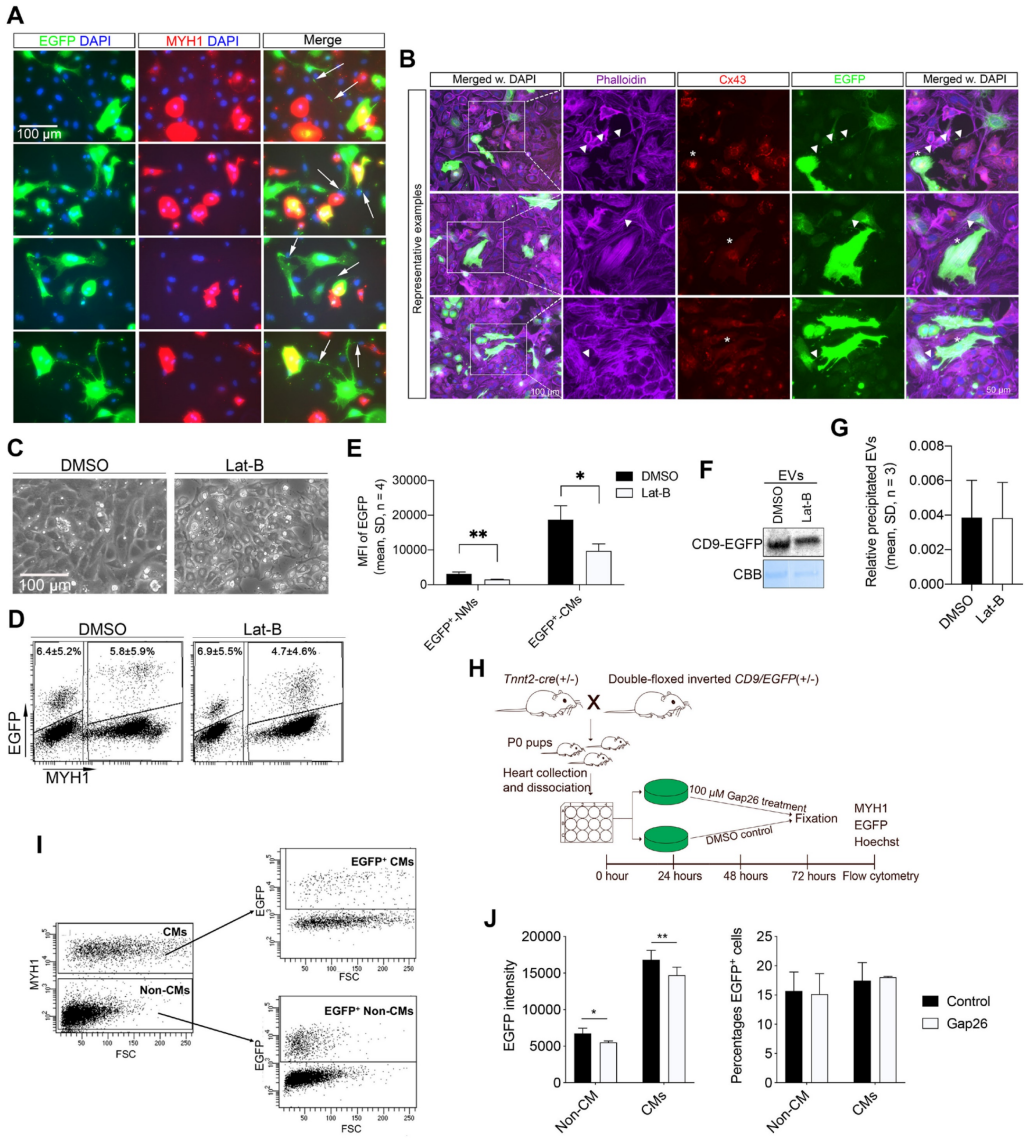
** Neonatal cardiomyocyte-derived EVs are transferred via type I tunneling nanotubes (TNTs) to neighboring cardiac cells.** (**A**) Representative close-up images from immunofluorescence staining of neonatal EGFP^+^ cardiac cultures for EGFP (green), MYH1 (red), and DAPI (blue). Exposure time was specifically increased to enable the visualization of tunneling nanotubes (arrows). This consequently, overexposed and blurred the EGFP signal in the body of the cells. (**B**) Immunofluorescence staining of neonatal EGFP^+^ cardiac cultures for EGFP (green), Phalloidin/Actin (purple), Cx43 (red), and DAPI (blue). Exposure time was specifically increased to enable the visualization of tunneling nanotubes. This consequently, overexposed and blurred the EGFP signal in the body of the cells. (**C-G**) (**C**) EGFP^+^ primary neonatal cardiac cells were cultured for 20 h with 1.25 µM Latrunculin B (Lat-B) or DMSO (control) and analyzed by flow cytometry for EGFP and MYH1 to determine the (**D**) percentage of EGFP^+^ cells and (**E**) the mean EGFP fluorescence intensity. For each experiment (n), n* = 8 animals were used. (**F**) Western blotting and (**G**) quantification of precipitated EGFP-labeled EVs from cell culture media after DMSO or 1.25 µM Lat-B treatment of EGFP^+^ cardiac cultures. For each experiment (n), n* = 5-7 animals were used. NMs: Non-myocytes, CMs: Cardiomyocytes. (**H-J**) (**H**) Schematic study design with two groups of primary neonatal cardiac cells i) Gap26 treated and ii) Control for 48h. (**I**) Representative flow cytometry plots of EGFP and MYH1 expression in the two groups of primary neonatal cardiac cells. (**J**) The mean EGFP fluorescence intensity and the percentage of EGFP^+^ cells. Data are shown as the mean ± SD. *P*-values were calculated using paired two-tailed Student's t-test or Two-way ANOVA with Fisher's LSD. ns, not significant * p < 0.05; **p < 0.01; *** p < 0.001.

TNTs are thin membranous tubes composed of either actin, tubulin, or both polymers that are known to interconnect cells, allowing direct communication between cells 42, 43. Due to the thin diameter of the TNTs, we had to increase the exposure time and magnifications during imaging to enable the detection of low-intensity EGFP signals within the TNTs of the neonatal EGFP^+^ cardiomyocytes *in vitro*. Accordingly, several intercellular nanotubes containing EGFP-labeled EVs were observed between EGFP^+^ cardiomyocytes and neighboring cardiac cells (Figure 4A). The nanotube structures observed herein protruded from the EGFP^+^ cardiomyocytes and attached to both neighboring cardiomyocytes and non-myocytes of a non-immediate EGFP origin (Figure 4A). The EGFP^+^ TNTs were phalloidin-positive (Figure 4B), indicating that transfer of neonatal cardiomyocyte-derived EVs occurs through type I TNTs consisting of F-Actin 43. To confirm the active transfer of EGFP^+^ EVs between neonatal cardiac cells, we exploited time-lapse imaging. From that, we found clear TNTs linking EGFP^+^ cardiomyocytes with recipient cells of EGFP^-^ origin, where EGFP signals travelled inside and along the TNT ending up at the junction of the recipient cells (Movie S1). These observations suggest that neonatal cardiomyocyte-derived EVs at least to some extent utilize TNTs as highways for intercellular transport, whereas secretion to the surroundings with subsequent uptake is thought to be the primary transfer mechanism hereto 18-20 seems limited. A few compounds such as Latrunculin B (Lat-B) have been shown to block type I TNT mediated transport by inhibiting the actin polymerization of the TNT. Our pre-experiments showed that adding Lat-B too early results in significant cell detachment and/or death of the primary cardiac cells. We therefore added Lat-B or DMSO vehicle (as control) to EGFP^+^ cultures 24 h after establishment and then tested whether this affected EGFP uptake in the neonatal cardiac cells (Figures 4C-G). After 20 h of TNT inhibition, we found a significant reduction in the fluorescence intensity of the EGFP signal in non-myocytes (Figure 4E) as well as in cardiomyocytes (Figure 4E). As expected, the percentage of EGFP^+^ cardiomyocytes (4.7 ± 4.6% vs. 5.8 ± 5.9%, p = 0.20, n = 4) and EGFP^+^ non-myocytes (6.9 ± 5.5% vs. 6.4 ± 5.2%, p = 0.06, n = 4) were similar between Lat-B and controls (Figure 4D) since TNT mediated EGFP^+^ EV transfer was enabled both *in vivo* before cell isolation and during the first 24 h of culture prior to TNT inhibition. Notably, we did not observe any difference in the overall amount of produced EGFP-labeled EVs in the EGFP^+^ cultures (Figures 4F-G), excluding that Lat-B treatment had impaired the production of EGFP-labeled EVs *in vitro*. Finally, we tested whether the type I TNT mediated neonatal cardiomyocyte EV transport also involved connexin 43 (Cx43) gap junctions that are known to play important roles in intercellular communication between cardiac cells 44, 45. Cx43 was indeed highly expressed in the main body of the EGFP^+^ cardiomyocytes but was absent in the protruding TNT (Figure 4B), the EGFP has been overexposed to enable visibility of the EGFP signals in the TNTs, due to their thin diameter. Thus, to evaluate whether Cx43 gap junctions participate in neonatal cardiomyocyte-derived EV transfer, we used a Cx43 mimetic peptide, Gap26, which specifically blocks Cx43 gap junctions 46, 47 (Figure 4H). Gap26 or vehicle (controls) were added to neonatal EGFP^+^ cultures 24 h after establishment and then analyzed by flow cytometry after an additional 48 h (Figure 4I-J). As such, the percentage of EGFP^+^ cardiomyocytes (17.5 ± 3.1% vs. 18.0 ± 0.2%, p > 0.05) and EGFP^+^ non-myocytes (15.7 ± 3.3% vs. 15.1 ± 3.5%, p > 0.05) were similar between vehicle and Gap26 treatments (Figure 4J), but the fluorescence intensity of the EGFP signal was significantly (p < 0.05) but only slightly reduced both in cardiomyocytes and non-myocytes (Figure 4J).

Together these data support that cardiomyocyte-derived EVs at the neonatal stage are transferred to neighboring cardiac cells through type I TNTs, and that Cx43 gap junctions are involved at some level in this transport.

### Cardiomyocyte-derived EVs are not cleared from the surroundings by macrophages but merely transferred via cell-cell interaction

**Figure 5:**
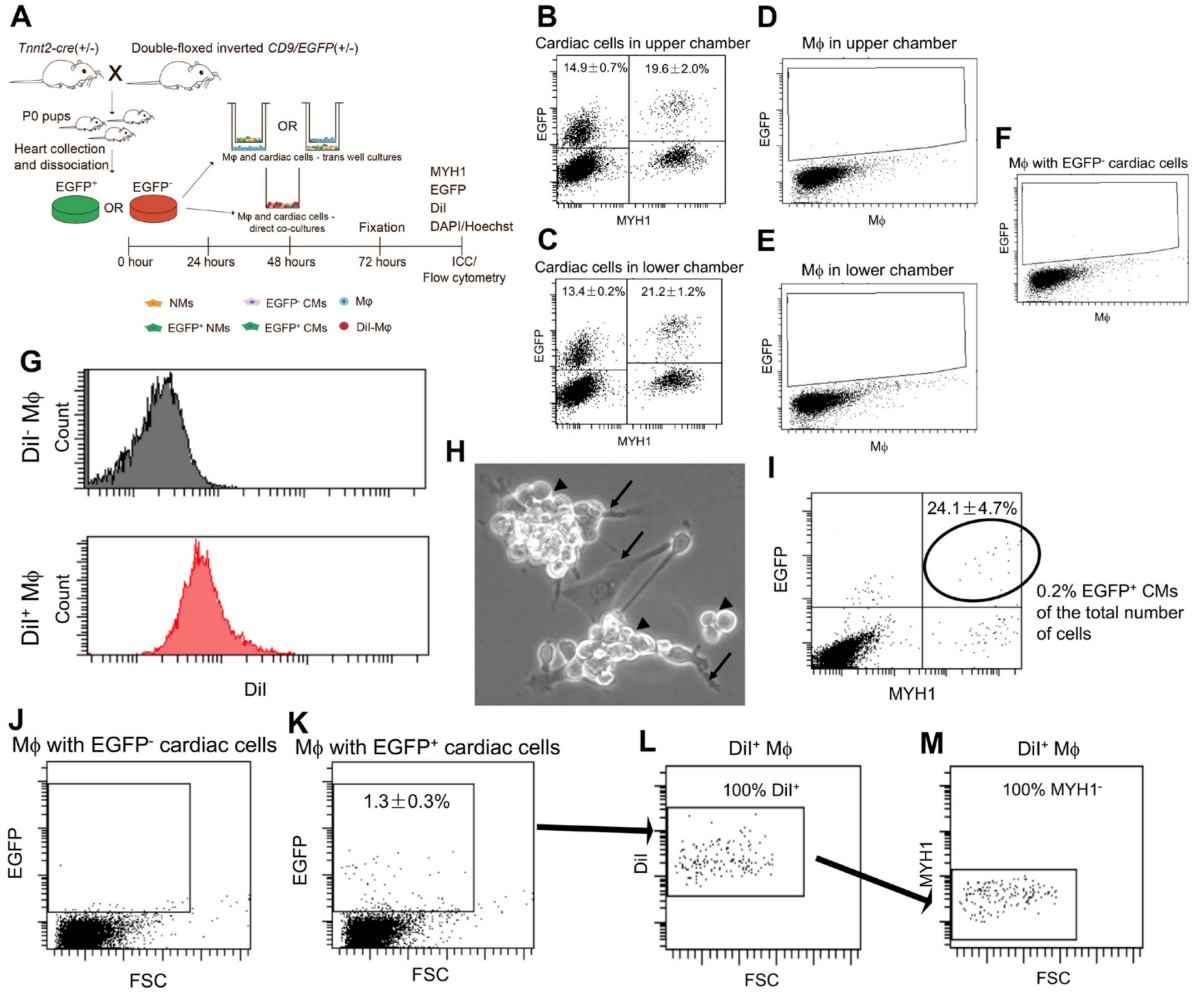
** Neonatal cardiomyocyte-derived EVs are transferred to macrophages (Mφ) via direct cell-cell contact, but not through the surroundings.** (**A**) Schematic study design of indirect (B-F) and direct (H-M) co-cultures between primary neonatal cardiac cells and macrophages (Mφ). (**B-C**) Representative flow cytometry plots of EGFP and MYH1 expression in cardiac cells (**B**) in inserts and (**C**) wells. (**D-E**) Representative flow cytometry plots of EGFP expression in Mφ (**D**) in wells and (**E**) inserts, in Mφ cultured with EGFP^+^ cardiac cells in B-C. (**F**) Representative flow cytometry plot of Mφ cultured with EGFP^-^ cardiac cells for control. (**G**) Representative flow cytometry histograms of DiI fluorescence in unlabelled (DiI^-^ Mφ) and DiI labelled (DiI^+^ Mφ) Mφ confirming that the entire population has increased fluorescence intensity (~100% DiI labelling efficiency before direct co-culture). (**H**) Representative close-up phase image of direct co-cultured cardiac cells (arrows) and Mφ (arrowheads). (**I**) Representative flow cytometry plot of EGFP and MYH1 expression in direct co-cultured cardiac cells and Mφ. (**J-K**) Representative flow cytometry plots of EGFP expression in Mφ directly co-cultured with EGFP^-^ (**J**) and EGFP^+^ cardiac cells (**K**). (**L-M**) Representative flow cytometry plots of the EGFP^+^ Mφ cells in (J) for DiI labelling (**L**) and absence of MYH1 (**M**) confirming their origin as Mφ. Percentages represent Mean ± SD (for indirect co-cultures n = 3 and for direct co-cultures n = 2).

Since none of our data supported that extracellular cardiomyocyte-derived EVs were taken up from the surroundings, we speculated whether secreted cardiomyocyte-derived EVs represent waste instead of signalling entities. This would agree with a recent study in the zebrafish showing that the majority of cardiomyocyte-derived EVs are cleared by macrophages 48, which are known to remove endogenous waste products. To test this in the neonatal mouse heart, we performed direct and indirect co-cultures of *Tnnt2-Cre*; double-floxed inverted *CD9/EGFP* positive (EGFP^+^) and their negative (EGFP^-^) cardiac cells with mouse macrophages (Figure 5A). For indirect cocultures, the neonatal EGFP^+^ cardiac cultures represented EGFP^+^ cardiomyocytes (Lower chamber: 21.2 ± 1.2% (mean, SD, n = 3) and Upper chamber: 19.6 ± 2.0% (mean, SD, n = 3)) and EGFP^+^ non-myocytes (Lower chamber: 13.4 ± 0.2% (mean, SD, n = 3) and Upper chamber: 14.9 ± 0.7% (mean, SD, n = 3)) (Figures 5B-C) which is in agreement with the above results (Figure 3). However, no immediate EGFP signals were detected in macrophages independent of location in the indirect cocultures (Figure 5D-F). Thus, despite macrophages being directly exposed to cardiomyocyte-derived EGFP^+^ EV conditioned medium they did not take up cardiomyocyte-derived EGFP^+^ EVs, at least not at a detectable level. Then to enable direct cocultures of neonatal EGFP^+^ cardiac cultures with macrophages and distinguish the macrophages in the co-cultures, we DiI labelled the macrophages (Figure 5A) with high efficiency (Figure 5G). After 72h of co-culture between neonatal EGFP^+^ cardiac cultures and DiI^+^ macrophages, microscopic examination showed a massive number of macrophages on top of the neonatal cardiac cells (Figure 5H). Flow cytometry confirmed that macrophages were in high excess in the co-culture likely due to a continued proliferation (Figure 5I) and at termination the percentage of EGFP^+^ cardiomyocytes only embraced around 0.2% of the entire co-culture (Figure 5H). Despite this low number of EGFP^+^ cardiomyocytes, we identified a clearly defined population of EGFP^+^ macrophages in the co-cultures (Figure 5J-K) and verified that they were DiI^+^/MYH1^-^ confirming their macrophage origin (Figure 5L-M). This suggested that the macrophages were able to take up cardiomyocyte-derived EGFP^+^ EVs upon direct cell-cell contact, but argued against the possibility that macrophages as a waste mechanism take up cardiomyocyte-derived EGFP^+^ EVs from the surroundings as otherwise seen in the zebrafish heart 48.

## Discussion

In the current study, we demonstrated by genetic tagging the *in vivo* transfer of neonatal cardiomyocyte-derived EVs to other cells but found it restricted to those cells within the neonatal heart. This was supported *in vitro* where the transfer of cardiomyocyte-derived EVs to non-myocytes and neighboring cardiomyocytes required direct cell-to-cell contact by type I TNTs in which the transfer occurs. While major research efforts have been made to characterize and use extracellular EVs for treatment, our results suggest that a significant part of biological intercellular EV signaling at least at the neonatal stage may occur without EV release to the extracellular medium.

Previous studies have suggested that cardiomyocyte-derived EVs could be diffused through the medium and taken up by non-myocytes 18, 33, 49. However, unlike these studies, we did not isolate and purify cardiomyocyte-derived EVs before being added to the target cell culture. Instead, we tagged the EVs using transgenic mice which allowed us to mimic the *in vivo* conditions more closely. Especially the contrasting concentrations of cardiomyocyte-derived EVs used between these two strategies may explain the discrepancy, where the use of extreme non-biological EV concentrations dominates the EV field in general. However, it is also possible that EV transfer occurs differently at different ages. Supportive of that, we did observe increasing percentages *in vivo* from P0 to P8 of non-myocytes having taken up cardiomyocyte-derived EGFP^+^ EVs, but this percentage was very low at P28 despite using a mouse line with higher labelling efficiency. Yet, in the co-cultures by sharing the EVs in the medium but preventing physical contact between the cells in our trans-well system, we excluded an effect from exaggerated EV concentrations and observed only transfer between neonatal cardiomyocytes and non-myocytes in the same chamber, but not between cells from different chambers. This was also confirmed by using macrophages in direct co-cultures. Previous studies have demonstrated that the effects of EVs are dose-dependent 50, 51, and concerns have been expressed as some studies use a higher concentration of EVs during *in vitro* experiments as compared to *in vivo* 21. By using the nano-luciferase-CD63 based EV reporter model developed by Luo et al. 22 investigators reported that cardiomyocyte-derived EVs are taken up by the kidney and lung in the adult mouse. Our results in the neonatal mouse do not support this observation and while we cannot exclude that cardiac-derived EVs circulate in the blood through the kidneys and the lungs, we note that the nano-luciferase-CD63 reporter does not distinguish the subcellular distribution of the EV as our CD9-EGFP mice. Notably, Nano-luciferase needs to react with the Nano-luciferase substrate before bioluminescence reading 22. Hence in the nano-luciferase-CD63 reporter, also the uptake *in vivo* and *in vitro* in cardiac fibroblasts requires fibroblasts and EVs to be isolated before being analyzed 22, respectively. This clearly includes the risk of luciferase-expressing cardiomyocyte contaminants in the isolated fibroblasts 52 and as discussed above non-physiological EV concentrations during *in vitro* experiments.

Considering previously demonstrated TNT cargoes and biology, the transfer of cardiomyocyte-derived EVs through TNT as observed herein, is somewhat not surprising. Type I TNTs are actin-based membrane tubes that connect cells, vesicles, or bacteria, and were first observed between non-contact liposomes 53-56 but subsequently demonstrated to mediate intercellular communication through transport of cargo between distant cells 57-60. From several recent studies, it is clear that nanotubes and EVs are important mediators of intercellular communication in the heart 61-63, and that these modes of communication are not mutually exclusive. Whereas others have shown that TNTs mediate the rapid transfer of mitochondria and inflammasomes between cardiomyocytes and non-myocytes in the heart 64, 65, we show that this should entail EVs as well. Interestingly, both studies 64, 65 as well as ours exploit neonatal mouse cardiomyocytes supporting that TNT mediated transport might be a general transport mechanism at the neonatal stage at least. Supportive of such a mechanism, a few *in vitro* data using isolated tumor EVs have been suggested to utilize TNTs for intercellular transport 40, 41, but this has not been confirmed by transgenic tagging.

TNTs can be classified into two types based on their formation. Type I TNTs contain actin filaments, the formation of which is driven by actin polymerization, while Type II TNTs are drawn-out when contacting cells separate and contain cytokeratin or tubulin filaments 43, 60. In our study, although the formation process of TNTs was not studied, inhibition of cardiomyocyte-derived EVs transfer by reduced actin polymerization suggests that neonatal cardiomyocyte-derived EVs are transferred through Type I TNTs. This was further evidenced by the fact that TNTs were phalloidin-positive. Moreover, the EGFP signals moving along the TNTs were similar to the gondolas observed in previous studies that were only present in type I TNTs 55, 60. Thus, *in vitro* where TNTs can be demonstrated, neonatal cardiomyocyte-derived EV transfer seems to be mediated through type I TNTs, and although the scenario may be different, these data may likely be recapitulated *in vivo*.

The type I TNTs were in our study negative for Cx43 gap junctions that otherwise allow direct transfer of molecules and ions up to approximately 1.2 kDa (~11 amino acids) 66, 67. Yet, it is known that Cx43 may reside in the EVs themselves and that the Cx43 gap junctions contribute to more efficient and rapid communication between EVs and recipient cells 68-70. For instance, previous studies have shown that Cx43 channels in cardiomyocyte-derived EVs will open after docking with Cx43 hemichannels at the plasma membrane of recipient cells and release cargos in a controlled manner 69, 70. In our study, the involvement of a Cx43 mediated mechanism was limited, but seems to occur at some level. Whether other cell-cell contact mechanisms besides the TNT and Cx43 described paths, are involved in the cardiomyocyte-derived EV transfer remains at this stage unknown.

Other than their luminal cargo, EVs also exert their functions through surface protein signaling 71. Previous study has shown that the vast majority of miRNA in cells, ranging from 96.2% to 99.9% of the total miRNA, is secreted in the non-EV fraction 72. This suggests that EVs may primarily mediate intercellular communication via ligand-receptor interactions. Therefore, it is worth noting that we cannot rule out that cardiomyocyte-derived EVs secreted into the cell surroundings may exert their biological functions by ligand-receptor binding on the cell surface.

Second, although tetraspanins CD63, CD9, and CD81 are all universal markers for cardiomyocyte-derived EVs 33, 73, we cannot exclude that our data exploiting CD9-EGFP as a marker for cardiomyocyte-derived EVs are limited to CD9-enriched cardiomyocyte-derived EVs. Moreover, we cannot rule out the possibility that cardiac cells may take up cardiomyocyte-derived EVs when supplied at extreme concentrations to the medium.

Despite these limitations, it seems reasonable from our data to speculate that extracellular shedded EVs at least in the neonatal heart are part of a waste mechanism rather than being part of intercellular signaling mediated by intracellular EVs through nanotube transport. Yet, since we did not observe macrophage uptake 48 of the secreted cardiomyocyte-derived EVs, it seems more likely that these EVs are destined for clearance by the kidney or liver. Whatever the final destination of extracellular EVs, the data could indicate that the extracellular and intercellular EV unities are quite different. This will be interesting to unlock in the future if techniques become available to distinguish between “waste and signaling EVs”, if they exist as such. Likewise, it will be critical to demonstrate if TNT mediated EV transfer is a fundamental mechanism of EV transport between cells that is present also in the adult heart and in other organs as well. If so, this may have a rather large impact on how we intend to exploit extracellular secreted EVs for regenerative medicine as currently suggested for a variety of organs/diseases 74-80.

In conclusion, we have developed a new genetic cardiomyocyte EV tagging approach, and by proof-of-concept have shed new light on EV biology in the neonatal heart demonstrating that cardiomyocyte-derived EVs mainly transfer via type I TNTs to neighboring cardiac cells. This suggests that intracellular EVs and TNTs may represent crucial coordinators of intercellular communication in the neonatal heart, whereas extracellular EVs may be less important at the neonatal stage of heart development.

## Materials and methods

### Animals

Different transgenic mouse lines were used in this study and housed with a 12/12 hour light/dark cycle. Firstly, *Tnnt2-cre* mice, also known as *cTnT-Cre* mice, which express Cre recombinase under the control of the rat troponin T2 (Tnnt2), cardiac promoter, were purchased from the Jackson Laboratory (stock no. 024240). Secondly, *αMHC-MerCreMer* mice, exhibiting tamoxifen-inducible Cre recombinase expression from the cardiac-specific alpha-myosin heavy chain (αMHC) promoter, were also purchased from the Jackson Laboratory (Stock No: 005657). Thirdly, we most recently generated double-floxed inverted *CD9/EGFP* mice in which CD9-EGFP expression is activated upon crossbreeding with Cre recombinase mice 26. By crossbreeding we then obtained *Tnnt2-Cre*; double-floxed inverted *CD9/EGFP* and *αMHC-MerCreMer;* double-floxed inverted* CD9/EGFP* mice, respectively. For Cre activation in the double heterozygous *αMHC-MerCreMer;* double-floxed inverted* CD9/EGFP* mice, we prepared Tamoxifen (Sigma-Aldrich, cat. no.: T5648) in corn oil (Sigma-Aldrich, cat. no.: C8267) with a concentration of 6 mg/mL, this was injected i.p. into neonatal P5 mice for four consecutive days at 40 mg/kg body weight/day 29, 81. Mice were sacrificed at three different postnatal stages (P0, P8, P28) and organs were harvested for further experiments including genotyping. All animal experiments were approved by the Danish Council for Supervision with Experimental Animals (#2016-15-0201-00941 and #2021-15-0201-01026).

### Genotyping

Recombined allele genotyping was performed through quantitative PCR high-resolution melting (qPCR-HRM) curve analysis with the genotyping primers listed in Table S2. The crude DNA was prepared from mouse tail or ear biopsies by incubation with alkaline lysis buffer and qPCR-HRM curve analysis was run on an Applied Biosystems QuantStudio 7 Flex Real-Time PCR System.

For isolation of genomic DNA, the interphase and organic phase from the initial homogenate with Tri Reagent were used as described 82. In brief, the DNA was precipitated with ethanol, washed by 0.1 M trisodium citrate in 10% ethanol followed by 75% ethanol, and finally dissolved in 8 mM NaOH. The pH of the isolated DNA was adjusted to 8.4 using HEPES before PCR amplification. PCR was carried out with Taq DNA polymerase (Thermo Fisher, cat. no.: 18038067) and the results were analyzed by agarose gel electrophoresis. The primers for transgene activation identification were PSV0504: *5′ GCAACGTGCTGGTTATTGTG 3′*, PSV0505: *5′ GCTGTACAAGGCAGCAAATG 3′*, and PSV0506: *5′ GTCCAATAGCAAGCACTGCA 3′.*

### Isolation of mouse cardiac cells

The mouse pups were sacrificed at P0 or P8 via decapitation, and the hearts were rapidly removed and placed in 100 µl ice-cold cardioplegic buffer (MIB; 1.2 mM KH_2_PO_4_ (pH 7.4); 0.25 g/L Na_2_CO_3_; 6.44 g/L NaCl; 2.6 mM KCl; 1.2 mM Mg_2_SO_4_; 11 mM glucose) supplemented with 1% (w/v) BSA (Sigma-Aldrich, cat. no.: A7888). Hearts were then dissociated using the Neonatal Heart Dissociation Kit (Miltenyi, cat. no.: 130-098-373) according to the manufacturer's instructions. After dissociation into a single cell suspension, cells were used for culture or fixed for flow cytometry.

P28 mice were sacrificed by cervical dislocation and the heart was excised and cut into small pieces in ice-cold perfusion buffer (120 mM NaCl, 14 mM KCl, 0.6 mM KH_2_PO_4_, 0.6 mM Na_2_HPO_4_, 1.2 mM MgSO_4_, 10 mM HEPES, 4.5 mM NaHCO_3_, 30 mM Taurine, 10 mM BDM, 5.5 mM glucose), and then digested with 0.5 mg/mL Protease XXIV (Sigma-Aldrich, cat. no.: P8038) for 12 min. Subsequently, the hearts were digested with 1160 units/mL collagenase type 2 (Worthington Biochemical Corporation, cat. no.: LS004177) in perfusion buffer supplemented with 5 µM CaCl_2_ for 35 min. The digestion was stopped by adding calf serum (Sigma-Aldrich, cat. no.: N4637) to a final concentration of 5% when all large heart tissue pieces were dispersed. Finally, the cell suspension was filtered through a 300 µm pore filter and fixed for flow cytometry.

### Medium exchange studies

Harvested cells were resuspended in culture medium (79.5% DMEM (Gibco, cat. no.: 10566016), 19.5% Medium 199 (Gibco, cat. no.: 11150059), and 1% FBS (Sigma-Aldrich, cat. no.: F0804)) supplemented with 1% penicillin/streptomycin (PS, Lonza, cat. no.: DE17-602E) and seeded on extracellular matrix (ECM, Sigma-Aldrich, cat. no.: E1270) pre-coated 12-well plates (one heart per well) for culturing (37°C and 5% CO_2_). After 24 h, the cells were defined as EGFP positive (EGFP^+^ cells) and EGFP negative (EGFP^-^ cells), based on the presence of EGFP. EGFP^-^ cells were randomly divided into two groups either receiving culture medium (EGFP^-^/control) or culture medium mixed with conditioned medium from EGFP^+^ cells (EGFP^-^/mixed medium). The culture medium was replaced daily, and the mixed medium was prepared daily by mixing filtered conditioned EGFP^+^ medium with fresh culture medium 1:1. After 96 h, the cultures were terminated, and cells were harvested for flow cytometry or fixed for ICC.

### Trans-well co-cultures

P0 hearts were dissociated individually and cells from one given heart were seeded in ECM pre-coated cell culture inserts with 0.4 μm pore size (Thermo Fisher, cat. no.: 140620) or separately in 24-well Carrier Plate (Thermo Fisher, cat. no.: 141008). This was done to avoid any growth of cardiac cells on the backside of the upper chamber which otherwise could connect with the cardiac cells in the upper chamber. After 24 h^83^, ^84^ unattached cells were removed, and EGFP^+^ cardiac cells were placed in the upper and EGFP^-^ cardiac cells in the lower chamber or vice versa using fresh wells. EGFP^-^ cardiac cells were placed in both upper and lower chambers as a negative control. The cultures were terminated after 96 h and cells were harvested for flow cytometry.

### Co-cultures using Raw 246.7

The macrophage cell line Raw246.7 (Mφ, a kind gift from Professor Daniel F. J. Ketelhuth, University of Southern Denmark) was cultured according to the manufacturer's recommendations (79 % DMEM (Lonza, cat. no.: BE12-604F/U1), 10% FBS supplemented with 1% penicillin/streptomycin). For trans-well co-cultures with Mφ, P0 hearts were processed as described above and the cells were seeded either in the upper chamber (insert) or lower chamber (well) as indicated. In the opposite part of the system, 150,000 Mφ were seeded. The cells were cultured in culture medium for primary cardiac cells. The cultures were terminated after 72 h and cells were harvested for flow cytometry. For direct co-cultures using Mφ, P0 hearts were processed as described above and the cells were seeded in ECM-coated wells and allowed to settle. Three hours after 150,000 DiI-marked Mφ were added to the wells. Beforehand, Mφ were marked with the CellTracker^TM^ CM-DiI (Invitrogen, cat. no.: C7000) using the manufacturer's recommended. The direct co-cultures were terminated after 72 h and cells were harvested for flow cytometry.

### Latrunculin B (Lat-B) treatment

P0 hearts pooled from a given litter were dissociated and seeded on ECM pre-coated 12-well plates. After 24 h, the F-actin polymerization inhibitor Lat-B (1.25 µM in dimethyl sulfoxide (DMSO, Sigma-Aldrich, cat. no.: D2650), Sigma-Aldrich, cat. no.: L5288) or DMSO alone was added to the cultures. The cells were treated for 20 h before harvesting for flow cytometry and the culture medium was collected for western blotting (WB) analysis.

### Gap26 trifluoroacetate inhibition

P0 mouse pups were sacrificed, and hearts were checked for the presence of EGFP^+^ cells under the microscope, before dissociation and culture. After 24 h, vehicle control (DMSO) or 0.1 mM Gap26 trifluoroacetate (Sigma-Aldrich, cat. no.: SML3074), a known inhibitor of connexin 43 (Cx43) was added to the medium, which was then refreshed every 24 h until analysis.

### Measurements of EV size and concentration

To enable determination of EV size during culture, the FBS fraction was ultra-centrifugated at 120,000 g at 4 ºC for 18 h before adding to the medium.

EV size and concentration were measured using a qNano Gold TRPS measurement system with a NP150 Nanopore (Izon Science, Oxford, United Kingdom) together with polystyrene calibration CPC100 beads (Izon Science, Oxford, United Kingdom). Medium samples were concentrated using ultrafiltration (Amicon® Ultra-4 Centrifugal Filter Device, Millipore, Darmstadt, Germany) and purified through a qEVoriginal/35 nm SMART column (Izon Science, Oxford, UK) before qNano quantifications according to manufacturer's instruction.

### Extracellular vesicle (EV) isolation and western blotting (WB)

EVs from the culture medium were isolated via polyethylene glycol (PEG) 6000 (Sigma-Aldrich, cat. no.: 81260). Briefly, the culture medium was centrifuged at 5,000 g for 15 min at 4 °C to remove cells and debris. Then an equal volume of 16% (w/v) PEG 6000 and 1 M NaCl solution was added to the culture medium while rotating overnight at 4 °C. The mixture was centrifuged at 5,000 g for 15 min at 4 °C and harvested EVs were resuspended in 100 µL RIPA-buffer (Millipore, cat. no.: 20-188). NuPAGE LDS Sample Buffer (4x) (Invitrogen, cat. no.: NP0007) and NuPAGE Sample Reducing Agent (10x) (Invitrogen, cat. no.: NP0009) were added to each sample, which was then heated at 95 °C for 10 min. 10 µl of samples were loaded on 15% SDS-PAGE gels (Bio-Rad, cat. no.: 4561086) and separated by electrophoresis, and the proteins were transferred to a PVDF membrane (Millipore, cat. no.: IPVH00010) by wet transfer. Blotted membranes were blocked for 1 h in 5% skim milk (Sigma-Aldrich, cat. no.: 70166) in Tris-buffered saline with Tween 20 (TBST; 20 mM Tris, 150 mM NaCl, and 0.05% Tween 20, pH 7.6) and CD9-EGFP protein was detected with GFP antibody (1:1000, GeneTex, cat. no.: GTX113617) followed by secondary goat anti-rabbit HRP conjugated antibody (1:2000, Dako, cat. no.: P0448). Coomassie brilliant blue (CBB) staining of the membranes was used as a loading control.

### Living cell confocal microscopy

The culture medium was switched to Hanks Balanced Salt Solution (HBSS; Lonza, cat. no.: BE10-527F) supplemented with 20 mM HEPES (Gibco, cat. no.: 15630056) and 2 g/L D-glucose (Sigma-Aldrich, cat. no.: G7021) before imaging. Living cell confocal microscopy was performed using the Olympus FV1000MPE Multiphoton Fluorescence Microscope system.

### RNA isolation and quantitative real-time PCR (qRT-PCR)

Total RNA was isolated using Tri Reagent (Thermo Fisher, cat. no.: AM9738) as previously described 85. Briefly, tissues were homogenized in 1 mL Tri Reagent using a gentleMACS Dissociator (Miltenyi Biotec), and 1-Bromo-3-chloropropane was used for phase separation. RNA was precipitated using 2-propanol, then rinsed in 75% ice cold ethanol, and finally dissolved in nuclease-free water. RNA concentration and purity were assessed using a nano-drop (Thermo Scientific). Purified RNA was reverse transcribed using the High-Capacity cDNA Reverse Transcriptase kit (Applied Biosystems, cat. no.: 4368814) according to the manufacturer's recommendation. Quantitative real-time PCR (qRT-PCR) was run on an Applied Biosystems QuantStudio 7 Flex Real-Time PCR System using Power SYBR Green PCR master mix (Applied Biosystems, cat. no.: 4367659). qRT-PCR data analysis was processed by the qbase^+^ platform (version 3.2; Biogazelle) using normalization against multiple stably expressed control genes as generally recommended to obtain robust qRT-PCR data 86. Briefly, the stability of each control gene was assessed by calculating the gene stability value (M) and coefficient of variation (CV) in qbase^+^ platform. The thresholds for the M and CV values were set at 0.5 and 0.2, respectively. Primers used for qRT-PCR are listed in Table S3.

### Immunohisto- and cytochemistry

For immunohistochemistry (IHC), tissues were embedded and snap-frozen in Tissue Tek (Sakura, cat. no.: 4583,). Ten μm thick cryosections were fixed in 10% neutral buffered formalin (NBF, Sigma-Aldrich, cat. no.: HT501128) for 10 min at room temperature (RT). Next, sections were either permeabilized and blocked in 0.05% Triton™ X-100 (Sigma-Aldrich, cat. no.: T8787)/2% Bovine serum albumin (BSA, VWR, cat. no.: 0332)/Tris-buffered saline (TBS) for 20 min or blocked in 2% BSA/TBS for 10 min. Primary antibody incubation was performed overnight at 4 °C followed by corresponding secondary antibody incubation for 1 h at RT. DAPI (Vector Laboratories, cat. no.: H-1200) was used to stain the nuclei.

For immunocytochemistry (ICC), cells were also fixed in 10% NBF for 10 min at RT prior to permeabilizing and blocking in 0.05% Triton™ X-100/2% BSA/TBS for 10 min. Cells were incubated with primary antibodies overnight (4 °C) or with Phalloidin for 1h followed by incubation with secondary antibodies (1 h, RT). Mounting was performed with DAPI and microscopic examinations with a Leica DMI4000B Cool Fluo Package instrument equipped with a Leica DFC340 FX Digital Camera. All antibodies used are listed in Table S4.

### Flow cytometry

As previously described 87, detached- or dissociated cells were fixed in 2.5% NBF diluted in HBSS/5%FBS/1%PS for 15 min at RT. After rinsing twice in HBSS/5%FBS/1%PS, cells were stored in HBSS/5%FBS/1%PS containing 0.05% sodium azide (NaN_3_) at 4 °C until further analysis. Fixed cells were stained with primary antibodies and visualized by corresponding secondary antibodies. The antibodies used are listed in Table S4. Hoechst 33342 (Sigma-Aldrich, cat. no.: B2261) was added 5 min before flow cytometry analysis (LSRII; BD Biosciences) and data were collected using the FACSDiva^TM^ software (version 8.0.1; BD Biosciences).

### Statistical analyses

All data and analysis consist of at least three independent experiments designated n, and when indicated n* refers to the number of animals in each experiment. Statistical significance of the difference between means was determined by either paired two-tailed Student's t-test or by one or two-way ANOVA followed by Dunnett's or Fisher's or Sidak post-tests as indicated. When applicable, the choice of the test included a normality check of data. The GraphPad Prism (9.0 Mac version) software was used for all statistical calculations. *p < 0.05, **p < 0.01, ***p < 0.001, ****p < 0.0001, ns (not significant).

## Supplementary Material

Supplementary figures and tables, movie legend.

Supplementary movie.

## Abbreviations

αMHC: alpha-myosin heavy chain
BSA: bovine serum albumin
CBB: coomassie brilliant blue
CMs: cardiomyocytes
Cx43: connexin 43
EGFP: enhanced green fluorescent protein
EV: extracellular vesicle
HBSS: hanks balanced salt solution
IHC: immunohistochemistry
Lat-B: latrunculin B
MVBs: multivesicular bodies
NaN_3_: sodium azide
NBF: neutral buffered formalin
NMs: non-myocytes
qRT-PCR: quantitative real-time PCR
RT: room temperature
TBS: tris-buffered saline
Tnnt2: troponin T2
TNTs: tunneling nanotubes
WB: western blotting
