# Supplementary material for: Transfer of cardiomyocyte-derived extracellular vesicles to neighboring cardiac cells requires tunneling nanotubes during heart development
Source: Theranostics. 2024 Jun 17;14(10):3843–58. doi: 10.7150/thno.91604 (PMC11234280; doi:10.7150/thno.91604)
Supplement: Supplementary file 1 — Supplementary figures and tables, movie legend. [file thnov14p3843s1.pdf]

Supplemental Information

Transfer of cardiomyocyte-derived extracellular vesicles to neighboring cardiac cells requires tunneling nanotubes during heart development

Ting Chen et al.

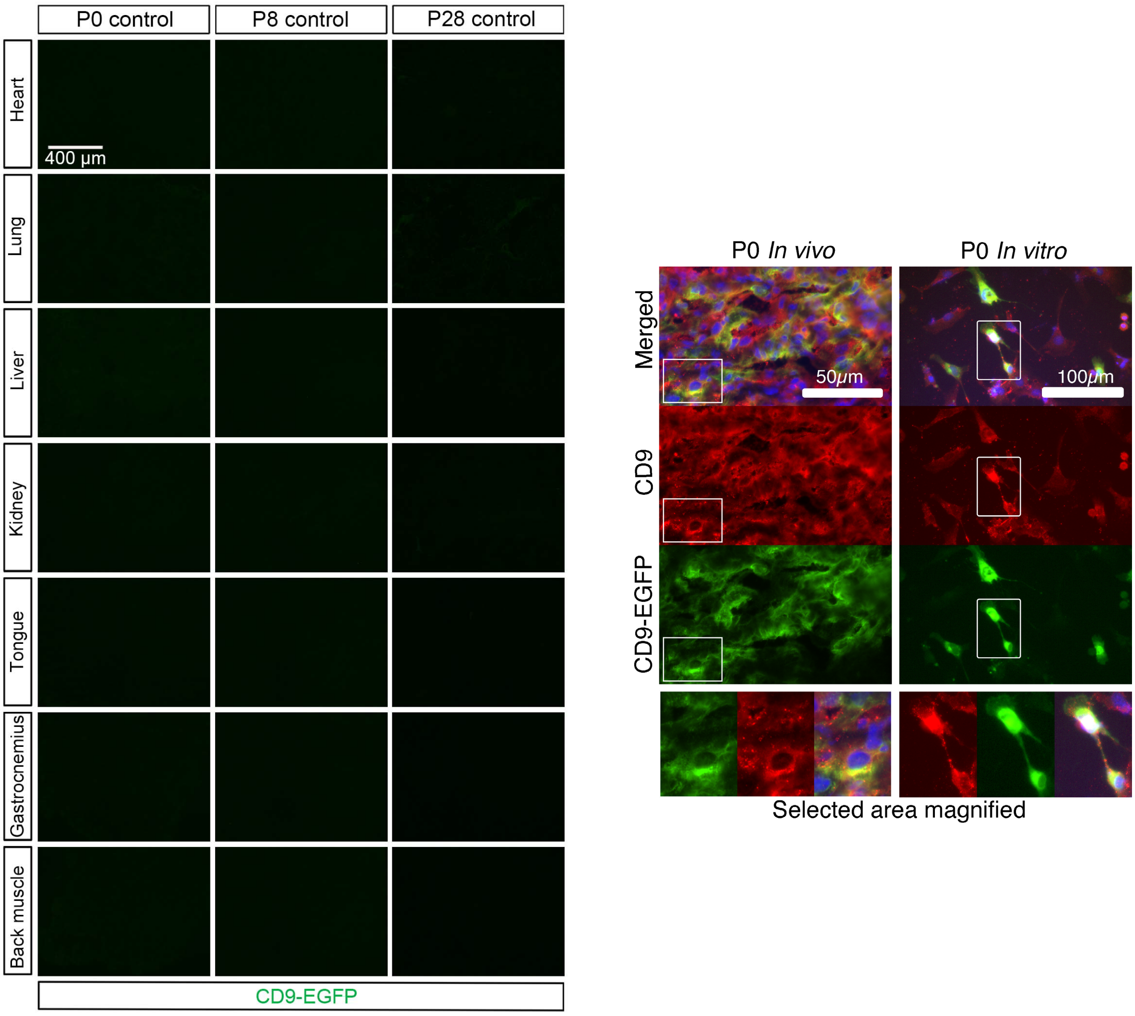

Figure S1. Direct fluorescence of different organs sections from P0, P8, and P28 *Tnnt2-Cre*; double-floxed inverted *CD9/EGFP*$^{-}$ mice and double staining with CD9 and EGFP both *in vitro* and *in vivo*.

No EGFP signal was detected in different organs sections from P0, P8, and P28 *Tnnt2-Cre*; double-floxed inverted *CD9/EGFP* $^{-}$ mice. Double staining with CD9 and EGPF show co-localization both *in vitro* and in *vivo* in *CD9/EGFP*$^{+}$ mice.

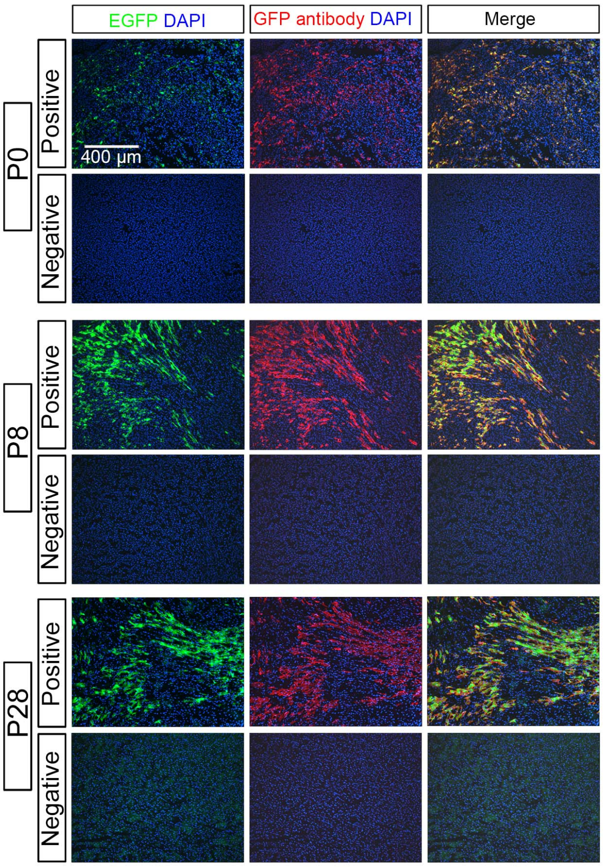

Figure S2. GFP antibody was used to amplify EGFP signals.

Immunofluorescence staining of heart sections from P0, P8, and P28 *Tnnt2-Cre*; double-floxed inverted *CD9/EGFP* $^{+}$ and $^{–}$ mice The GFP antibody signals (red) were co-localized with the EGFP signals (green). Positive: *Tnnt2-Cre*; double-floxed inverted *CD9/EGFP* $^{+}$ mice, Negative: *Tnnt2-Cre*; double-floxed inverted *CD9/EGFP* $^{-}$ mice.

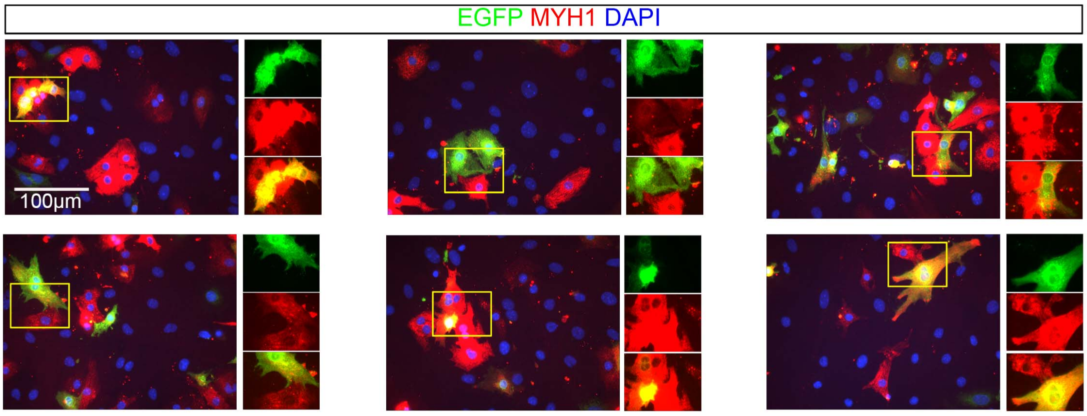

Figure S3. EGFP-labeled EVs were present in a fraction of cardiomyocytes.

Immunofluorescence staining of $EGFP^+$ cardiac cultures for EGFP (green), MYH1 (red), and DAPI (blue) (n = 4).

Table S1. The pregnant *αMHC-MerCreMer* mouse exhibited poor outcomes after tamoxifen administration.

Mouse ID	Methods	Outcome
1	Injecting tamoxifen 50 mg/kg body weight/day to pregnant mice for 2 consecutive days.	Stillbirth
2		Dystocia
3	Injecting tamoxifen 20 mg/kg body weight/day to pregnant mice for 4 consecutive days from E14-E17.	Dystocia
4		Dystocia
5		Dystocia

Table S2. List of genotyping primers used in qPCR-HRM curve analysis.

Gene	Primer	Sequence
Cre	Cre Trangene F	GCGGTCTGGCAGTAAAAACTATC
	Cre Trangene R	GTGAAACAGCATTGCTGTCACTT
CD9-EGFP	EGFP F	AAGCTGACCCTGAAGTTCATCTGC
	EGFP R	CTTGTAGTTGCCGTCGTCCTTGAA
Interleukin-2	Internal Ctrl F	CTAGGCCACAGAATTGAAAGATCT
	Internal Ctrl R	GTAGGTGGAAATTCTAGCATCATCC

Table S3. List of qRT-PCR primers used in this study.

Gene	Primer	Sequence
CD9-EGFP	PSV 0504	GCAACGTGCTGGTTATTGTG
	PSV 0506	GTCCAATAGCAAGCACTGCA
Beta-actin	Beta-actin F	GCTGTATTCCCCTCCATCGTG
	Beta-actin R	CACGGTTGGCCTTAGGGTTCAG
RPL13A	RPL13A F	CGAAGATGGCGGAGGGGCAG
	RPL13A R	ACCACCACCTTCCGGCCCAG

Table S4. List of antibodies used in this study.

Antibody	Manufacturer, catalog #	Host species	Dilution
GFP	Abcam (Cat. no.: ab290)	Rabbit	1:500 (IHC, ICC, Flow)
GFP	Abcam (Cat. no.: ab13970)	Chicken	1:500 (IHC)
MYH1	DSHB (Clone MF20)	Mouse	1:200 (IHC, ICC), 1:300 (Flow)
GFP	GeneTex(Cat. no.: GTX113617)	Rabbit	1:1000 (WB)
CD31	BD Pharmingen (Cat. no.: 553370)	Rat	1:50 (IHC)
Vimentin	Abcam (Cat. no.: ab45939)	Rabbit	1:500 (IHC)
Phalloidin	Invitrogen (Cat. no.: A30107)	Amanita phalloides	1:800 (IHC)
Connexin 43	Sigma-Aldrich (Cat. no.: SAB4200730)	Mouse	1:250 (IHC)
Donkey anti-Rabbit IgG, Alexa Fluor 488	Invitrogen (Cat. no.: A-21206)	Donkey	1:200 (IHC, ICC, Flow)
Goat anti-Chicken IgY, Alexa Fluor 488	Invitrogen (Cat. no.: A-11039)	Goat	1:200 (IHC)
Donkey anti-Rabbit IgG, Alexa Fluor 555	Invitrogen (Cat. no.: A-31572)	Donkey	1:200 (IHC)
Donkey anti-Mouse IgG, Alexa Fluor 555	Invitrogen (Cat. no.: A-31570)	Donkey	1:200 (IHC, ICC, Flow)
Goat anti-Rat IgG, Alexa Fluor 555	Invitrogen (Cat. no.: A-21434)	Goat	1:200 (IHC)
Donkey anti-Mouse IgG, Alexa Fluor 647	Invitrogen (Cat. no.: A-31571)	Donkey	1:200 (Flow)
Goat Anti-Rabbit Immunoglobulins/HRP	Dako (Cat. no.: P0448)	Goat	1:2000 (WB)

Movie S1. Time-lapse microscopic imaging of tunneling nanotubes linking $EGFP^+$ cardiomyocyte with a recipient cell of $EGFP^-$ origin.

EGFP signals appeared along the nanotube and at the junction of the recipient cell.
